# Proteomic Investigations of Lysine Acetylation Identify Diverse Substrates of Mitochondrial Deacetylase Sirt3

**DOI:** 10.1371/journal.pone.0050545

**Published:** 2012-12-07

**Authors:** Eri Maria Sol, Sebastian A. Wagner, Brian T. Weinert, Amit Kumar, Hyun-Seok Kim, Chu-Xia Deng, Chunaram Choudhary

**Affiliations:** 1 Department of Proteomics, The Novo Nordisk Foundation Center for Protein Research, Faculty of Health Sciences, University of Copenhagen, Copenhagen, Denmark; 2 Genetics of Development and Disease Branch, The National Institute of Diabetes and Digestive and Kidney Diseases, National Institutes of Health, Bethesda, Maryland, United States of America; 3 Department of Life Science, Division of Life and Pharmaceutical Sciences, College of Natural Science, Ewha Womans University, Seoul, Republic of Korea; Ludwig-Maximilians-Universität München, Germany

## Abstract

Lysine acetylation is a posttranslational modification that is dynamically regulated by the activity of acetyltransferases and deacetylases. The human and mouse genomes encode 18 different lysine deacetylases (KDACs) which are key regulators of many cellular processes. Identifying substrates of KDACs and pinpointing the regulated acetylation sites on target proteins may provide important information about the molecular basis of their functions. Here we apply quantitative proteomics to identify endogenous substrates of the mitochondrial deacetylase Sirtuin 3 (Sirt3) by comparing site-specific acetylation in wild-type murine embryonic fibroblasts to Sirt3 knockout cells. We confirm Sirt3-regulated acetylation of several mitochondrial proteins in human cells by comparing acetylation in U2OS cells overexpressing Sirt3 to U2OS cells in which Sirt3 expression was reduced by shRNA. Our data demonstrate that ablation of Sirt3 significantly increases acetylation at dozens of sites on mitochondrial proteins. Substrates of Sirt3 are implicated in various metabolic pathways, including fatty acid metabolism and the tricarboxylic acid cycle. These results imply broader regulatory roles of Sirt3 in the mitochondria by modulating acetylation on diverse substrates. The experimental strategy described here is generic and can be applied to identify endogenous substrates of other lysine deacetylases.

## Introduction

Lysine acetylation is a reversible posttranslational modification (PTM) that occurs on proteins involved in the regulation of diverse cellular processes, including mitochondrial functions [1], [2]. Acetylation is dynamically regulated by lysine acetyltransferases (KATs, also known as histone acetyltransferases or HATs), and by lysine deacetylases (KDACs, also known as histone deacetylases or HDACs) [3]. Lysine acetylation is evolutionary conserved from bacteria to humans, suggesting that its regulatory scope is of ancient origin.

Despite important regulatory functions of acetylation, until recently only a limited number of endogenous acetylation sites were known. Owing to the extreme complexity of mammalian proteomes, and possibly low stoichiometry of modified sites, mapping endogenous acetylation sites has been a challenging task. Using antibody-based affinity enrichment, a proteomic survey discovered nearly 300 acetylation sites on mitochondrial proteins [4]. Recently, we applied high resolution mass spectrometry (MS) for mapping endogenous acetylation sites and identified 3,600 acetylation sites in human cells, of which over 500 sites were localized on mitochondrial proteins [5]. Considering that phosphorylation, the most extensively studied PTM in eukaryotic cells, is relatively sparse in this bacterially-derived organelle [6], [7], these numbers of acetylation sites are surprisingly large.

The human and mouse genomes each encodes 18 different KDACs, of which 11 are classified as zinc-dependent deacetylases [8]. The remaining seven are NAD^+^-dependent deacetylases, known as Sirtuin1–7 (Sirt1–7) [9]. Sirtuins are localized to specific sub-cellular compartments: Sirt3, 4, and 5 in the mitochondria, Sirt6 and 7 in the nucleus, and Sirt1 and 2 in both the cytoplasm and nucleus. Sirtuins are important regulators of mammalian physiology whose functional roles are believed to be conserved from yeast to mammals [9]–[11].

Among sirtuins, Sirt3 has emerged as a key regulator of mitochondrial physiology [12]–[14]. Sirt3 deficient mice appear phenotypically normal under non-stress conditions; however, acetylation of mitochondrial proteins is noticeably increased [15]. We have demonstrated that Sirt3 is an important regulator of the cellular energy homeostasis and that cellular ATP levels are markedly reduced in Sirt3 deficient mice [16]. Sirt3 functions as a tumor suppressor and maintains mitochondrial integrity during cellular stress [4]. In addition, several recent reports demonstrated the involvement of Sirt3 in diverse cellular processes such as fatty acid metabolism [12], oxidative stress response [17], tumor suppression [4], and age-associated hearing loss [13]. Despite the growing evidence on the regulatory roles of Sirt3 in cellular metabolism, an unbiased, site-specific analysis of its endogenous substrates is lacking. Considering the overall increased acetylation of mitochondrial proteins in Sirt3 deficient mice, we hypothesized that many more Sirt3-regulated acetylation sites in this organelle remain to be identified.

Here we apply SILAC-based quantitative proteomics to identify endogenous substrates of Sirt3 in murine and human cells. Our data show that Sirt3 modulates acetylation of more than a quarter of all mitochondrial acetylation sites quantified in this study. A majority of proteins with Sirt3-regulated acetylation sites are enzymes that participate in the regulation of metabolic pathways such as the tricarboxylic acid cycle (TCA) cycle, pyruvate metabolism, fatty acid metabolism, and branched-chain amino acid metabolism. The presented dataset substantially expands the number of known Sirt3 targets, providing a valuable resource for researchers aiming to understand the mechanisms by which Sirt3 regulates cellular physiology.

## Results

### Strategy for Sirt3-regulated acetylome analysis

**Figure 1:**
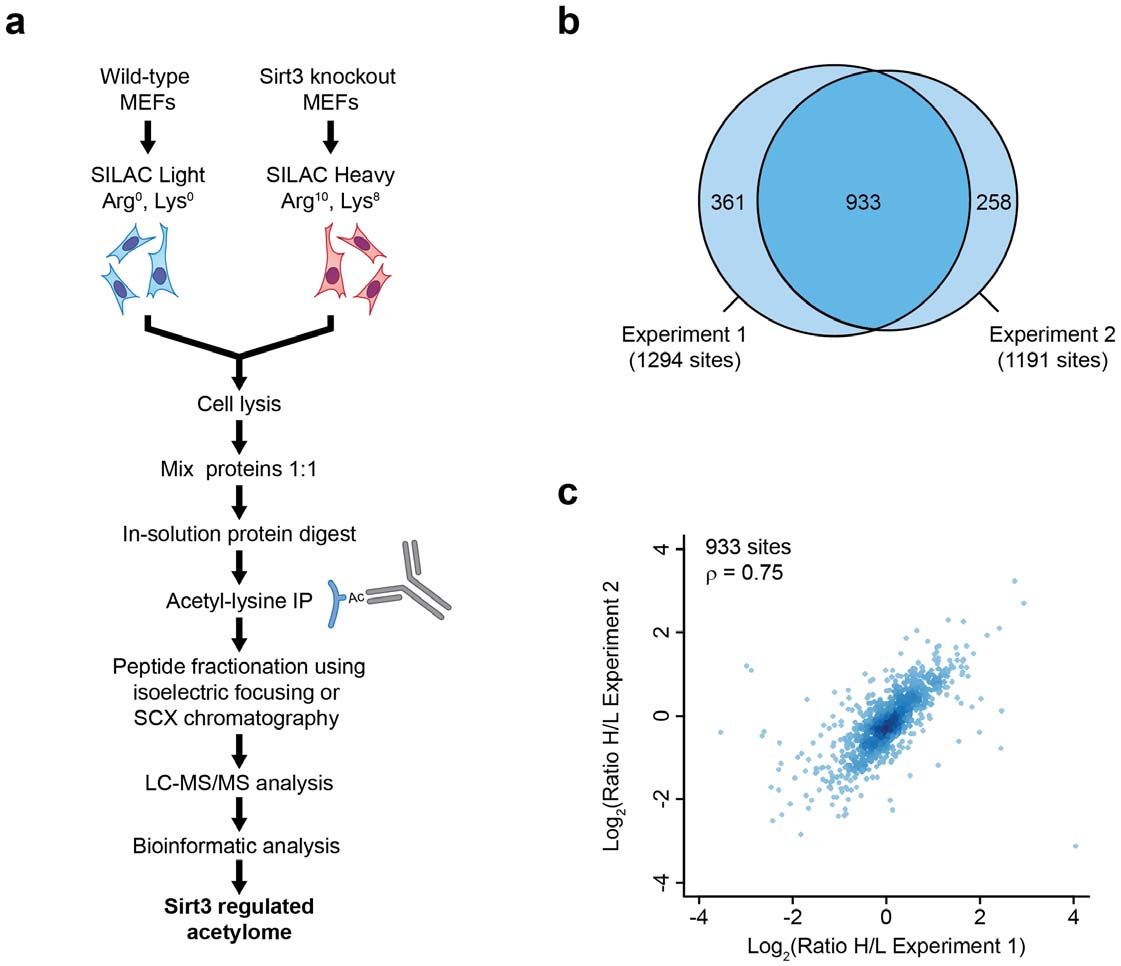
Experimental strategy for mapping of Sirt3-regulated acetylation sites. (A) SILAC-based proteomics strategy for quantification of Sirt3 regulated acetylation sites. Wild type mouse embryonic fibroblasts (MEFs) were labeled with light amino acids, whereas Sirt3 knockout MEFs were labeled with heavy amino acids. Lysine acetylation was analyzed as described previously [5]. (B) Overlap between the acetylation sites quantified in two independent experimental replicates. (C) Correlation of SILAC ratios determined in two biological replicates. Correlation was calculated using Pearson's correlation coefficient.

To identify substrates of Sirt3 in mammalian cells we used mouse embryonic fibroblasts (MEFs) from Sirt3 wild-type (WT) or Sirt3 knockout (KO) animals [16]. A stable isotope labeling by amino acids in cell culture (SILAC)-based quantitative proteomics strategy [18] was used to measure the differences in acetylation between Sirt3 WT and KO cells. Sirt3 wild-type cells were grown in ‘light’ SILAC media (L-arginine-^12^C_6_-^14^N_4_, and L-lysine-^12^C_6_-^14^N_2_) whereas Sirt3 knockout cells were cultured in ‘heavy’ SILAC media (L-arginine-^13^C_6_-^15^N_4_, and L-lysine-^13^C_6_-^15^N_2_) (Figure 1A). Cell lysates from the both SILAC cell populations were mixed in equal amounts, and proteins were digested into peptides using trypsin. Acetylated peptides were enriched from the resulting complex peptide mixture with an anti-acetyllysine antibody as described previously [5].

### Accurate mapping and quantification of acetylation sites

To identify in vivo lysine acetylation sites we used online liquid chromatography coupled tandem mass spectrometry (LC-MS/MS) on an Orbitrap instrument (LTQ-Orbitrap Velos or Q Exactive) [19], [20]. All peptides were sequenced using a ‘high-high strategy’ in which peptides were fragmented by ‘higher energy C-trap dissociation’ (HCD) and both the intact peptide ions and their fragment ions were measured in the high mass accuracy Orbitrap detector [21]. All resulting data were analyzed using MaxQuant software [22], allowing a maximum false discovery rate (FDR) of 1% at peptide and protein level. FDRs were estimated using a target-decoy strategy [23].

### Sirt3 is a key regulator of mitochondrial acetylation

**Figure 2:**
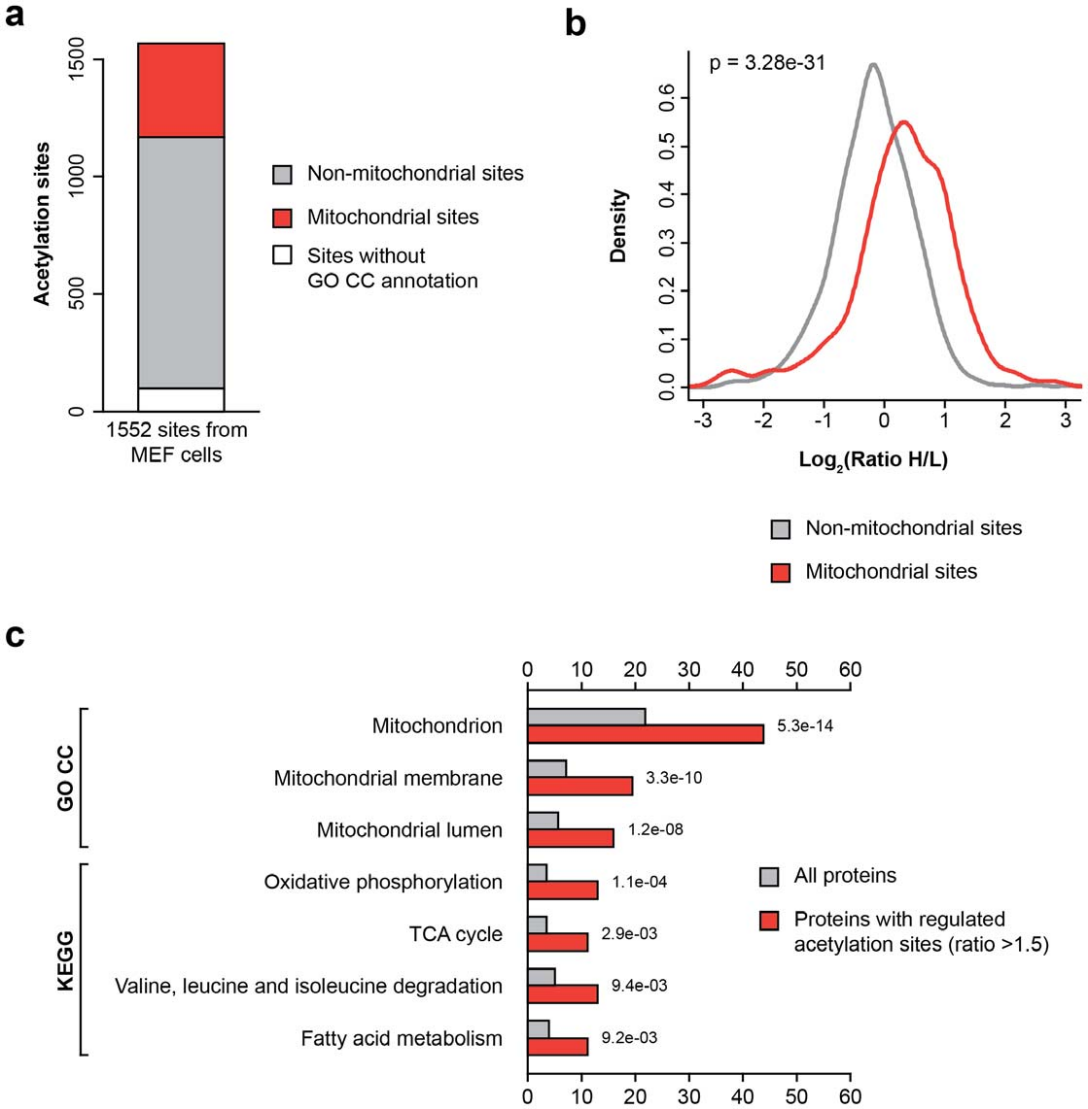
Cellular and functional annotations of Sirt3 regulated proteins. (A) Cellular distribution of identified acetylation sites. (B) Significantly elevated acetylation of mitochondrial sites in Sirt3 knockout cells. Logarithmized SILAC ratios of acetylated peptides from mitochondrial and non-mitochondrial proteins were plotted. The data shows the upwardly shifted distribution of SILAC ratios of mitochondrial acetylation sites in Sirt3 deficient cells. Statistical significance was calculated using Wilcoxon rank sum test. (C) Gene Ontology and KEGG pathway enrichment analysis of Sirt3-regulated proteins. Proteins with increased acetylation in Sirt3 knockout cells showed significant enrichment of several mitochondrial associated GOCC or KEGG terms.

In this study, we quantified over 1,500 acetylation sites in MEFs from two biological replicate experiments (Figure 1B, Table S1). About 60% of the reported acetylation sites were quantified in both replicate experiments with good reproducibility as determined by Spearman's rank correlation (ρ = 0.75) (Figure 1C). In accordance with previous studies that demonstrated extensive acetylation of mitochondrial proteins [5], [24], [25], a substantial portion of the identified acetylation sites were present on proteins annotated as mitochondrial (Figure 2A). Considering the mitochondrial localization of Sirt3, we hypothesized that acetylation sites on mitochondrial proteins should show increased acetylation in Sirt3 knockout cells. We plotted the logarithmized SILAC ratios of the quantified acetylation sites on mitochondrial and non-mitochondrial proteins (Figure 2B). In our experiments, cells deficient for the deacetylase activity are grown in ‘heavy’ SILAC media and control wild-type cells in ‘light’ SILAC media; therefore, Sirt3-regulated sites should show an increase in the heavy/light (H/L) SILAC ratios. As expected, these data showed that the distribution of SILAC ratios of mitochondrial acetylated peptides is shifted towards higher SILAC H/L ratios, demonstrating significantly increased (p = 3.28e-31) acetylation of mitochondrial proteins in the absence of Sirt3. Over one hundred acetylation sites showed more than 2-fold increase in Sirt3 knockout cells, a majority of these are located on mitochondrial proteins (Table S1). Overall, protein abundance levels were not substantially altered between the wild-type and Sirt3 knockout cells (Table S1); therefore, individual acetylation ratios provided here were not normalized for protein abundances.

We performed Gene Ontology (GO) term and KEGG pathway enrichment analysis to identify cellular compartments and biological pathways with significantly increased acetylation in Sirt3 knockout cells. Acetylation sites on proteins annotated with mitochondrial GO cellular compartment terms were significantly more frequently increased in acetylation. The same is true for proteins involved in several KEGG metabolic pathways such as fatty acid metabolism, leucine, isoleucine and valine degradation, and the tricarboxylic acid (TCA) cycle (Figure 2C). Our data indicates that Sirt3 mainly regulates acetylation of mitochondrial proteins that are involved in metabolic pathways. These findings are in agreement with the known localization and function of Sirt3 in the mitochondria.

### Identification of putative Sirt3 substrates in human cells

**Figure 3:**
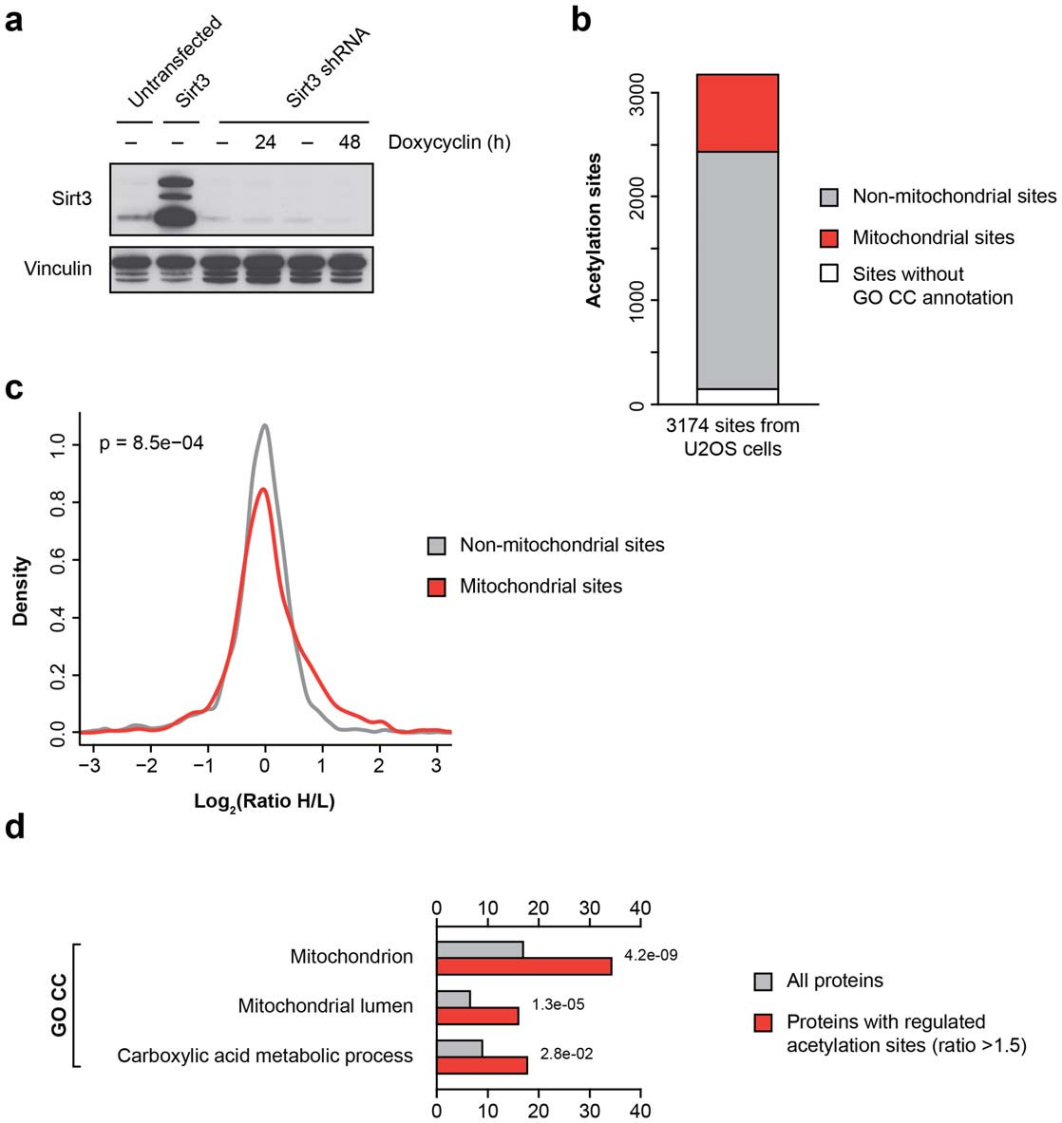
Identification of Sirt3-regulated acetylation sites in human cells. (A) Generation of Sirt3 knockdown and overexpression U2OS cells. U2OS cells were either transfected with a human Sirt3 encoding cDNA or with a Sirt3-specific shRNA. To induce expression of Sirt3-shRNA, cells were treated with doxycycline for the indicated time periods. Expression of Sirt3 was analyzed by immunostaining using anti-Sirt3 antibody. (B) Distribution of identified acetylation sites between mitochondrial or non-mitochondrial cellular compartments. (C) Increased acetylation of mitochondrial acetylation in Sirt3 knockdown cells. Logarithmized SILAC ratios of acetylated peptides from mitochondrial and non-mitochondrial proteins were plotted. These data shows the upwardly shifted distribution of SILAC ratios of mitochondrial acetylation sites in Sirt3 knockdown cells. (D) Gene Ontology enrichment analysis of Sirt3-regulated proteins. Proteins with increased acetylation in Sirt3 knockdown cells showed significant enrichment of mitochondrial associated GO terms.

To validate the results obtained from Sirt3 knockout mouse cells in a different organism, we created a model system based on U2OS cells (a human ostesarcoma cell line). In these cells we either increased cellular Sirt3 levels by retroviral overexpression of human Sirt3, or reduced its expression using an inducible shRNA-based knockdown approach. Overexpression and conditional knockdown of Sirt3 was confirmed at the protein level by Western blotting (Figure 3A). Sirt3 overexpressing cells were grown in ‘light’ SILAC media whereas Sirt3 knockdown cells were cultured in ‘heavy’ SILAC media and acetylation analysis was performed as described above (Figure 1A). Using this approach, we identified over 3,000 acetylation sites in human U2OS cells, of which about 23% were present on mitochondrial proteins (Figure 3B, Table S2). In agreement with the data obtained from Sirt3 knockout MEFs, acetylation of mitochondrial sites was significantly increased in comparison to non-mitochondrial acetylation sites (Figure 3C). Furthermore, analysis of proteins with increased acetylation in Sirt3 deficient cells revealed that mitochondria associated GO terms were enriched among Sirt3-regulated proteins (Figure 3D).

### Sirt3 modulates acetylation of proteins involved in diverse metabolic pathways

**Figure 4:**
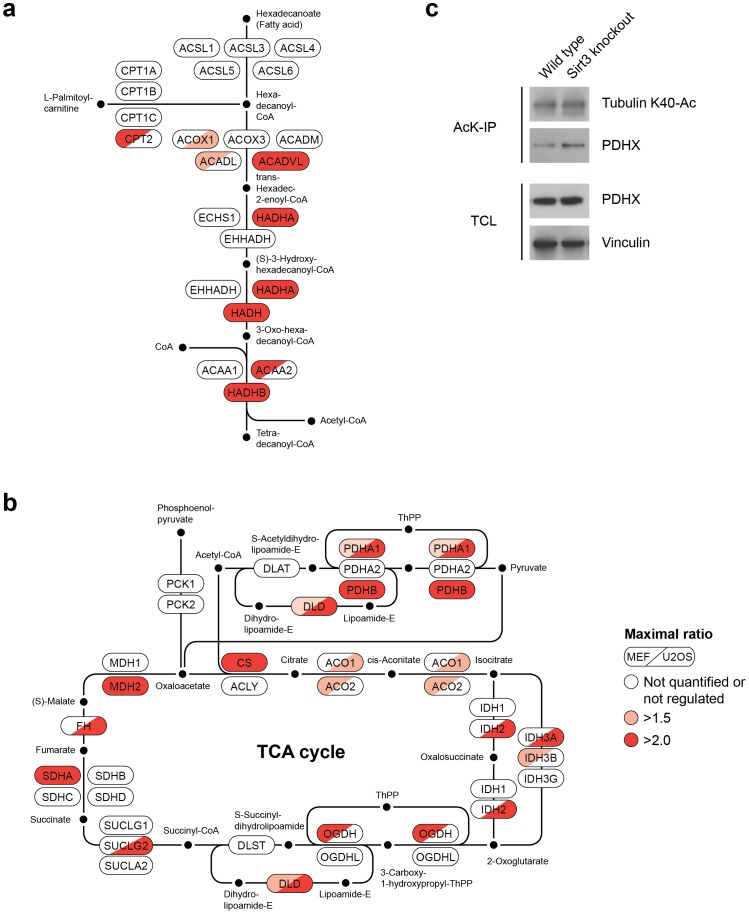
Sirt3 increases acetylation of enzymes involved in fatty acid metabolism and the TCA cycle. (A and B) The panel A shows enzymes involved in the fatty acid metabolism, and the panel B shows enzymes involved in the TCA cycle. Enzymes in these pathways are color coded (indicated at the top of the figure) based on their identification in our experiments, and their increase in acetylation in Sirt3 knockout or knockdown cells. (C) Acetylation of PDHX is increased in Sirt3 knockout cells. Acetylated proteins were immunoprecipitated using an anti-acetyllysine antibody and immunoblotted with anti-PDHX or anti-acetyltubulin antibodies. The lower panel shows protein levels of PDHX in whole cell lysates used as input for immunoprecipitations, and vinculin was used as loading control.

Deficiency of Sirt3 increases acetylation of several enzymes involved in metabolic pathways. For example, acetylation of several enzymes involved in the fatty acid metabolism (fatty acid elongation and beta-oxidation) is increased in cells with reduced Sirt3 activity (Figure 4A). Recently, it has been reported that Sirt3 deacetylates long-chain specific acyl-CoA dehydrogenase (LCAD) [12], an important regulator of fatty acid metabolism. Our data confirm increased acetylation of LCAD in Sirt3 deficient murine cells. We also find that acetylation of very long-chain specific acyl-CoA dehydrogenase (VLCAD) is increased upon Sirt3 knockdown in U2OS cells. Thus, we speculate that Sirt3 may also regulate function of VLCAD in human cells. In contrast to mouse cells, human cells predominantly express VLCAD [26], and it is indispensable for the β-oxidation of fatty acids in these cells.

Similarly, acetylation of proteins involved in the tricarboxylic acid (TCA) cycle is increased in Sirt3 deficient cells (Figure 4B). In contrast to fatty acid metabolism, the role of Sirt3 in this process is less clear. It has been reported that Sirt3 regulates the activity of succinate dehydrogenase A (SDHA, also known as SDH2), and increased acetylation of SDHA was observed in Sirt3 deficient cells with pan-anti-acetyllysine antibody staining in Western blotting experiments [27], [28]. Our data show that K177 of SDHA is a potential Sirt3 target site, and its regulation is conserved both in human and mouse cells. Interestingly, our data also showed that ablation of Sirt3 increased acetylation of several components of the pyruvate dehydgrogenase complex (Figure 4B), including PDHA, PDHB and DLD. Acetylation of PDHX, which is associated with this complex, was also increased in Sirt3 deficient cells. Sirt3-dependent acetylation of PDHX K394 was observed both in murine and human cells. The increased acetylation of this protein in Sirt3 knockout cells was confirmed by immunostaining (Figure 4C). It has been reported that acetylation of MDH2 can regulate its activity. However, the enzyme(s) involved in its deacetylation are not known. Our data now identify Sirt3 as putative deacetylase that regulates MDH2 acetylation in mammalian cells, and suggest that Sirt3 may regulate MDH2 function via lysine deacetylation.

## Discussion

Lysine deacetylases are key regulators of animal physiology [9], [29], and are attractive drug targets for treating human diseases such as cancer, diabetes, and neurodegerative disorders [3], [30]. Recent mass spectrometry-based studies have discovered a large number of acetylation sites [5], [7], [25], [31], [32]. Methods that identify substrates of specific deacetylases can help to illuminate the molecular mechanisms of these enzymes. Here we use genetically altered mammalian cells, and a SILAC-based quantitative proteomics screen, to identify in vivo substrates of Sirt3, a prominent deacetylase localized to the mitochondria. The described approach is generic and can easily be applied to identify substrates of other lysine deacetylases.

Mitochondrial metabolism is tightly linked to the internal energy state of the cell, which can be reversibly regulated in response to nutrients or environmental stress [33]. Acetylation has been suggested to be an anciently conserved regulatory mechanism that connects the cellular metabolism with the energy status of cells, and Sirt3 in particular has emerged as a key regulator of cellular energy homeostasis [16]. Previous work using genetic and biochemical techniques has shown that genetic ablation of Sirt3 results in increased acetylation of mitochondrial proteins [15]. However, an unbiased identification of Sirt3 target proteins and, more importantly, the precise mapping of the regulated acetyl-lysines on these targets had previously been challenging. Our data now confirms earlier findings at a site level and show that Sirt3 is a major regulator of mitochondrial acetylation. Ablation of Sirt3 increased acetylation of many mitochondrial proteins, although the overall increase in acetylation in cultured cells is relatively modest. Sirt3 is an important regulator of metabolic pathways at the organismal level and it is possible that many of Sirt3 substrates may not be expressed in these cells or not easily detectable in our approach. Nevertheless, we were able to confirm Sirt3-depedent acetylation of several previously known targets and identified many novel, putative substrates of this deacetylases.

Sirt3 has been shown to regulate fatty acid metabolism by deacetylation of LCAD in murine cells [12]. Here we find that Sirt3 deacetylates VLCAD in human cells. In addition, several other enzymes which participate in fatty acid elongation and β-oxidation are deacetylated by Sirt3 (Figure 4A). Based on in vitro assays it has already been postulated that Sirt3 may regulate acetylation of some of these enzymes [34], and our data now provides evidence that Sirt3 regulates these sites in vivo. Recently, it has been reported that Sirt3 may also control ketone body production [35] and amino acid catabolism [34]. Sirt3 may also play an important role in the detoxification of reactive oxygen species [17], as well as in the beneficial effects of calorie restriction - both mediated by deacetylation of the superoxide dismutase 2 (SOD2) enzyme. Several enzymes in the tricarboxylic acid cycle showed increased acetylation in Sirt3 knockdown cells (Figure 4B), therefore different steps of this pathway may also be regulated by Sirt3. In fact, while we were analyzing these data, IDH2 was identified as an important target of Sirt3 which protects cells from oxidative stress [13]. We also find Sirt3-dependent acetylation of this enzyme and identify K384 as an evolutionary conserved acetylation site that is controlled by this deacetylase. Based on the identification of several previously known, as well as novel putative Sirt3 substrates identified here, we speculate that Sirt3 may have a broader role in metabolic regulation. However, it remains to be determined whether acetylation of the additional putative Sirt3 substrates identified here has functional roles in metabolism, and if their functions in these processes are evolutionary conserved.

Our results demonstrate that combining genetic/molecular biology approaches with quantitative proteomics provides an elegant way to identify substrates of lysine deacetylases in cell lines. Genetic knockout cells can be used as a model system for identification of deacetylase substrates as illustrated by identification of several known substrates of Sirt3. We show that it is feasible to identify substrates of deacetylases by manipulating their expression levels by ectopic protein expression or gene knockdown by siRNA-based approaches. It is plausible that ectopic expression of deacetylases may result in deacetylation of non-native substrates, and may complicate the interpretation of results. Nevertheless, this approach can be useful for identification of putative substrates of lysine deacetylases or their validation in different model organisms, as we have shown here. This strategy is also applicable for discovering substrates of deacetylases that are important for cellular viability, and for which it is not possible to obtain complete knockout cells.

In summary, our proteomics screen identified a large number of endogenous substrates of Sirt3 in mammalian cells. Our results show that Sirt3 deacetylates a large number of enzymes involved in key metabolic pathways implying a broader regulatory role of Sirt3 in mitochondrial processes. These results exemplify the usefulness of combining genetic tools with quantitative MS screens to identify endogenous substrates of lysine deacetylases. The Sirt3 targets identified here may provide a starting point to analyze their functional relevance in cellular metabolism.

## Experimental Procedures

### Cell culture

MEFs were prepared from murine embryos (E12.5 or E13.5) derived from mating of Sirt3+/− and Sirt3+/− mice [4], and grown in DMEM containing 15% FBS. U2OS cells expressing murine ecotropic receptor [36] and Plat-E [37] cells were cultured in DMEM medium supplemented with 10% FBS, 2 mM L-glutamine, 1% penicillin and streptomycin. For SILAC labeling cells were cultured with either L-arginine and L-lysine or L-arginine-^13^C_6_-^15^N_4_ and L-lysine-^13^C_6_-^15^N_2_ (Cambridge Isotope Laboratories) as described previously [18]. All cells were cultured at 37°C in a humidified incubator containing 5% CO_2_.

### Generation of Sirt3 overexpression and knockdown U2OS cell lines

For generating stable Sirt3 overexpressing U2OS cell line, the full length Sirt3 cDNA was obtained from the Ultimate ORF cDNA clone collection (Invitrogen) and transferred into the retroviral expression vector pMY-IRES-EGFP using the Gateway technology (Invitrogen). U2OS cells expressing murine ecotropic receptor were transduced with Sirt3 containing retroviruses. Retrovirus production and target cell transduction was performed as described previously [38]. For generating a tet-repressor expressing stable U2OS cell line, we used an ecotropic receptor expressing U2OS cell line [39] and transfected with pCDNA6/TR plasmid (Invitrogen). Bulk cell population was selected using blasticidin. For generating the conditional Sirt3 knockdown cells, Sirt3 shRNA was cloned into pSUPERIOR-retro-puro plasmid (OligoEngine, Seattle, WA), a tetracycline-regulated retroviral shRNA expression vector. U2OS cells (expressing tet repressor) were retrovirally transduced with pSUPERIOR-retro-puro containing a Sirt3 specific shRNA sequence (GGACAGAAGAGATGCGGGAttcaagagaTCCCGCATCTCTTCTGTCC), and stably transduced cells were selected using puromycin. For Sirt3 knockdown experiments, cells were treated with doxycycline (1 µg/ml, Sigma-Aldrich) for 48 hours before cell lysis.

### Immunoprecipitation and immunoblotting

Immunoprecipitations were performed according to standard procedure with an anti-acetyllysine antibody conjugated to beaded agarose (Immunechem). Immune complexes were washed five times in ice-cold RIPA-buffer (1% NP-40, 1 mM sodium deoxycholate, 150 mM NaCl, 1 mM EDTA, 50 mM Tris, pH 7.5) with protease inhibitor (Complete protease inhibitor tablets, Roche Diagnostics) and eluted with 40 µl SDS sample buffer. Eluates were resolved by 4–12% gradient SDS-PAGE and transferred onto PVDF membrane. The membrane was blocked using 5% skimmed milk powder in PBS tween-20 (0.1%). PDHX was detected using an anti-PDHX antibody (ProteinTech) followed by incubation with Protein-G conjugated to horseradish peroxidase (Bio-Rad Laboratories). Sirt3-expression was analyzed by subjecting total cell lysate to SDS-PAGE and immunodetected using an anti-Sirt3 antibody (Cell Signaling Technology). As a loading control, an antibody against Vinculin (Sigma-Aldrich) or Acetyl-α-Tubulin (Cell Signaling Technology) were used. The secondary antibodies coupled to horseradish peroxidase (Jackson ImmunoResearch Laboratories) were used for immunodetection. The detection was performed with Novex ECL Chemiluminescent Substrate Reagent Kit (Invitrogen).

### MS sample preparation

Sirt3 knockout and wild-type control cells were cultured in SILAC media. Also, SILAC-labeled Sirt3 knockdown or overexpressing cells, U2OS cells expressing exogenous Sirt3 or U2OS expressing Sirt3 shRNA were cultured for 48 hours in the presence of 1 µg/ml doxycycline prior to lysis. Cells were washed twice with 1× phosphate buffered saline and lysed in RIPA-buffer containing 1% NP-40, 1 mM sodium deoxycholate, 150 mM NaCl, 1 mM EDTA, 50 mM Tris (pH 7.5), and protease inhibitor (Complete protease inhibitor tablets, Roche Diagnostics). Lysates were incubated for 15 minutes on ice, and were cleared by centrifugation (17,000 g, 15 minutes at 4 degrees). Protein concentration of the cleared lysates was measured with BCA Protein Assay Reagent (Thermo Scientific) and proteins from two SILAC populations were mixed 1∶1. Proteins from cell lysates were acetone precipitated by adding 4-fold excess acetone, and storing overnight at −20°C. The precipitated proteins were re-dissolved in 6M urea/2M thiourea/10 mM HEPES (pH 8), reduced with 1 mM dithiothreitol (DTT), alkylated with 5.5 mM chloroacetamide (CAA) [40] and subsequently digested with endoproteinase Lys-C and modified sequencing grade trypsin as described previously [40], [41]. Protease digestion was stopped by adding trifluoroacetic acid (TFA) to a final concentration of 1%, and precipitation was cleared by centrifugation. Peptides were purified using reversed-phase Sep-Pak C18 cartridges (Waters) and peptides were eluted in 50 percent acetonitrile, 0.1% TFA. The peptides were re-dissolved in immunoprecipitation (IAP) buffer (50 mM MOPS pH 7.2, 10 mM sodium phosphate, 50 mM sodium chloride) and incubated with an anti-acetyllysine antibody for 12 hours at 4°C on a rotation wheel, as described previously [5]. The immunoprecipitates were washed three times with IAP-buffer followed by additional 3 washes with distilled water. Residual water was removed and acetylated peptides bound to antibodies were eluted by 0.1% TFA in water.

### Fractionation of peptides and mass spectrometric analysis

The peptides from immunoaffinity purification were fractionated with isoelectric focusing [42], [43] using the Agilent 3100 OFFGEL Fractionator (Agilent) or using strong cation exchange-based microcolumns [44], [45]. Peptides were purified using reversed phase C18 micro StageTips as described previously [46]. The peptides were eluted from stage tips with 40 µl of 40% acetonitrile, 0.5% acetic acid into a 96 well plate. Acetonitrile was removed by speed-vac Concentrator Plus (Eppendorf) and the volume was reduced to ∼5 µl. Peptide fractions were analyzed on a LTQ-Orbitrap Velos or Q Exactive mass spectrometer (Thermo Scientific) equipped with an nanoflow HPLC system (Thermo Scientific) as described [19], [20], [47]. The MS was operated in data dependent mode to automatically switch between MS and MS/MS acquisition. Survey full scan MS spectra (from m/z 300–1700) were acquired in the Orbitrap analyzer, after accumulation to a target value of 1,000,000 ions. The ten most intense ions were sequentially isolated and fragmented by higher-energy C-trap dissociation (HCD) and fragment spectra recorded in the Orbitrap. FA ion from ambient air (m/z 445.120025) was used for internal calibration as described earlier [48]. Typical mass spectrometric conditions (for LTQ Orbirtrap Velos) were: spray voltage, 2.2 kV; no sheath and auxiliary gas flow; heated capillary temperature, 275°C. The ion selection threshold was 5,000 counts for HCD MS2. For HCD an isolation width of 4.0, an activation time of 0.1 ms, and normalized collision energy of 40% were used.

### Peptide identification and computational analysis

Raw data files were processed and analyzed using MaxQuant software (development version 1.2.2.9 [22]. Parent ion and MS2 spectra were searched against the mouse IPI protein database (version 3.68, 56,729 entries, combined with 248 common contaminants) or human IPI protein database (version 3.68, 87,061 entries, combined with 248 common contaminants) using the Andromeda search engine [49]. Spectra were searched with a mass tolerance of 6 ppm in MS mode, 20 ppm in HCD MS/MS mode, strict trypsin specificity, and allowing up to 2 missed cleavage sites. Minimum required peptide length was 6 amino acids. Cysteine carbamidomethylation was searched as a fixed modification, whereas N-acetyl protein, oxidized methionine and acetylation of lysine were searched as variable modifications. The false discovery rate (FDR) for peptides and sites was estimated using a target-decoy approach and fixed at one percent [23]. Acetyl-lysine site identifications were filtered to remove C-terminally located acetyl-lysines (assuming that acetylation of lysine blocks peptide cleavage by Lys-C and trypsin). Statistical analysis was performed using the R software environment. Annotation enrichment analysis was performed using the Database for Annotation, Visualization and Integrated Discovery (DAVID) [50]. Mapping of human and mouse acetylation sites was performed using orthology assignments and multi-sequence alignments from the eggNOG database version 3.0 [51]. All modified peptide sequences were mapped to eggNOG protein sequences and orthology groups. Lysine residues with the same position in the multi-sequence alignment were considered as corresponding lysine residues.

## Supporting Information

Table S1 **List of all acetylation sites quantified in Sirt3 knockout MEF cells.**
(XLSX)

Table S2 **List of all acetylation sites quantified in human U2OS Sirt3 knockdown cells.** **Column header descriptions for Tables S1, S2.** **Proteins:** Identifiers of all proteins that contain the acetylation site. **Leading proteins:** Identifiers of the best scoring proteins that contain acetylation the site.**Positions:** Position of the acetylation site for all leading proteins. **Protein:** Best scoring protein that contains the acetylation site. **Position:** Position of the acetylation for the best scoring protein. **Protein Names:** Names of all proteins that contain the acetylation site. **Gene Names:** Names of all genes associated with the proteins that contain the acetylation site. **Uniprot:** Uniprot database identifiers. **Localization probability:** Probability that modification occurs at the specified position in the peptide. **Sequence window:** Amino acid sequence flanking the acetylated lysine. **Modified sequence:** Peptide sequence with modifications, oxidation (ox) and acetylation (ac) indicated. **Localization (K) probabilities:** Probabilities for the localization of the acetyl group on all lysines in the peptide sequence. **Score:** Andromeda search engine peptide score. **PEP:** Posterior Error Probability of the identification. **Charge:** Charge state of the peptide. **m/z:** The mass-over-charge of the peptide. **Mass Error (ppm):** Mass error of the recalibrated mass-over-charge value of the precursor ion in comparison to the predicted monoisotopic mass of the identified peptide sequence. **Ratio H/L:** SILAC ratio for the acetylation sites.
(XLSX)
